# 

**Published:** 1871-01

**Authors:** 


### CONTENTS:

ORIGINAL COMMUNICATIONS:

ART. I. Cavernous Tumor of the Orbit, complicated with a large Sanguineous Cyst; successful Removal without injury to the Globe or Optic Nerve. By E. L. Holmes, M.D.... 1

ART. II. Chronic Pemphigus (Pompholix Diutinus of Willan and Bateman). By Walter Hay, M.D....... 4

ART. III. Cook Co. Hospital Clinic. Reported by C. T. Fenn, M.D ..... 8

ART. IV. Hydrate Chloral. By S. W. Gould, M.D..................... 12

ART. V. Trismus Nascentium. By Theo. W. Stull, M.D..... ......... 14

ART. VI. Case of Imperforate Anus, with Deformity of the Organs of Generation. By Moses Barrett, M.D. 15

ART. VII. Cases in Private Practice. By Jno. E. Owens, M.D............ 17

SELECTIONS ............................ 21
Palpitation: its Diagnostic Value. Judge Thayer's Charge in the case of Haire *vs.* Reese.

EDITORS' BOOK TABLE.................. 36
Galvano-Therapeutics; Physics and Physiology of Spiritualism; Specific Medication and Specific Medicines; Transactions of the Am. Med. Association; Treatise on Fractures and Dislocations.

PAMPHLET NOTICES...................... 40

NON-PROFESSIONAL EXCHANGES......... 43

EDITORIAL ............................. 44

MEDICAL ITEMS, NEWS, AND GOSSIP.... 49

LOOT ................................... 51

OBITUARY............................... 63

